# HucMSC‐exosomes carrying miR‐326 inhibit neddylation to relieve inflammatory bowel disease in mice

**DOI:** 10.1002/ctm2.113

**Published:** 2020-06-21

**Authors:** Gaoying Wang, Jintao Yuan, Xiu Cai, Zhiwei Xu, Jingyan Wang, Dickson K.W. Ocansey, Yongmin Yan, Hui Qian, Xu Zhang, Wenrong Xu, Fei Mao

**Affiliations:** ^1^ Key Laboratory of Medical Science and Laboratory Medicine of Jiangsu Province, School of Medicine Jiangsu University Zhenjiang Jiangsu P. R. China; ^2^ The People's Hospital of Danyang Affiliated Danyang Hospital of Nantong University Zhenjiang Jiangsu P. R. China

**Keywords:** hucMSC‐Ex, IBD, miR‐326, NEDD8, neddylation, NF‐κB

## Abstract

**Background:**

Inflammatory bowel disease (IBD) is a group of chronic intestinal inflammation that is a risk factor for many gastrointestinal cancers. Exosomes are gradually gaining attention as an emerging treatment method for IBD due to their important biological characteristics. NF‐κB is an important pro‐inflammatory transcription factor kept inactive by IκB protein in the cytoplasm by masking the nuclear localization signal of NF‐κB. The deterioration of IκB is mainly ubiquitination, and this depends on neddylation.

**Methods:**

In this study, we established a dextran sulfate sodium (DSS)‐induced IBD model in BABL/C mice to evaluate the effect of human umbilical cord mesenchymal stem cell‐derived exosomes (hucMSC‐exosomes, hucMSC‐Ex) on the repair of IBD. At the same time, human colorectal mucosa cells (FHC) were stimulated by LPS (lipopolysaccharide) in vitro to activate the inflammatory environment to study the mechanism of hucMSC‐Ex regulating neddylation. The microRNA (miRNA) obtained by sequencing and transfection with hucMSC‐Ex was used to verify the role of miR‐326/neddylation/IκB/NF‐κB signaling pathway in IBD repair.

**Results:**

HucMSC‐Ex inhibited the process of neddylation in relieving DSS‐induced IBD in mice. The binding of NEDD8 (neural precursor cell‐expressed, developmentally downregulated gene 8) to cullin 1 and the activation of NF‐κB signaling pathway were suppressed along with reduced expression levels of neddylation‐related enzyme molecules. The same phenomenon was observed in FHC cells. The miRNA comparison results showed that miR‐326 was highly expressed in hucMSC‐Ex and played an important role in inhibiting the neddylation process. The therapeutic effect of hucMSC‐Ex with high expression of miR‐326 on IBD mice was significantly stronger than that of ordinary hucMSC‐Ex.

**Conclusions:**

HucMSC‐Ex relieves DSS‐induced IBD in a mouse model by inhibiting neddylation through miR‐326.

## Graphical abstract

HucMSC‐Ex carrying miR‐326 relieves intestinal inflammatory damage by inhibiting neddylation within the intestinal mucosal cells. A, HucMSC‐Ex contains multiple active substances, of which miR‐326 is highly expressed. B, miR‐326 targets NEDD8 and inhibits the binding of cullin 1 and NEDD8 (the neddylation of cullin 1), thereby preventing the degradation of IκB; NF‐κB is masked, and the activation of its signaling pathway is suppressed to alleviate inflammation.

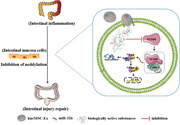

**Abbreviations**
CD: Crohn's disease
Co‐IP: coimmunoprecipitation
CRL: cullin‐RING ligase
DAI: disease activity index
DC: dendritic cell
DSS: dextran sulfate sodium salt
Evs: extracellular vesicles
FBS: fetal bovine serum
FHC: human colorectal mucosa cell
H&E: hematoxylin and eosin staining
HucMSC: human umbilical cord mesenchymal stem cell
HucMSC‐Ex: human umbilical cord mesenchymal stem cell‐derived exosomes
IBD: inflammatory bowel disease
IEC: intestinal epithelial cell
IF: immunofluorescence
IFN‐γ: interferon‐γ
IHC: immunohistochemistry
IκB: inhibitor of NF‐κB
LPS: lipopolysaccharide
MSC: mesenchymal stem cell
NEDD8: neural precursor cell expressed developmentally down‐regulated 8
NF‐κB: nuclear factor‐κB
NK: natural killer cell
PCNA: cell proliferation nuclear antigen
qRT‐PCR: real‐time fluorescence quantification PCR
TGF‐β: transforming growth factor‐β
TNBS: 2,4,6‐trinitrobenzene sulfonic acid
TNF‐α: tumor necrosis factor‐α
Treg: regulatory T cell
UC: ulcerative colitis

## 1 INTRODUCTION

Inflammatory bowel disease (IBD) is a group of inflammatory disorders caused by environmental, genetic, infection and immune factors. These factors interact to affect the intestinal mucosal immune system. IBD mainly includes ulcerative colitis (UC) and Crohn's disease (CD). The incidence of IBD has increased rapidly in recent years.^1^, ^2^ In patients with IBD, chronic intestinal inflammation is a major risk factor for the development of gastrointestinal cancer, including colon cancer, small intestinal adenocarcinoma, intestinal lymphoma, anal cancer, and bile duct cancer,^3^ hence IBD interventions can prevent the occurrence of gastrointestinal cancer.^4^ At present, despite the use of various protocols such as immunomodulators, thiopurine agents, and anti‐TNF monoclonal antibodies in the treatment of IBD, the therapeutic outcomes are still not satisfactory.^4^ There is a need for alternative methods in the clinical treatment of IBD.

Mesenchymal stem cells (MSCs) isolated from various tissues have the ability of inducing regeneration, maintaining general tissue dynamic balance, and home to target site.^5^ The role of MSCs in tissue repair has been addressed in a variety of disease models^6^, ^7^ with the report that their efficacy in improving the function of damaged tissues is mainly related to paracrine effects rather than direct implantation and differentiation.^8^, ^9^, ^10^ As one of the composite factors contributing to the paracrine effect of MSCs, extracellular vesicles (EVs) have recently been identified as new participants in cell‐to‐cell communication.^11^, ^12^ EVs are nano‐sized (40‐150 nm) membranous vesicles produced during the formation of multivesicular bodies (MVB)^13^, ^14^, ^15^, ^16^ and can transport lipids, mRNA and proteins, and alter cell behavior through paracrine or autocrine processes.^17^, ^18^ They mediate short‐ and long‐distance intercellular communication and deliver different types of biologically active substances to recipient cells.^19^, ^20^ Exosomal‐mediated pathway can enhance the antigen‐presenting capacity of intestinal epithelial cells (IEC). Compared with B cells and T cells, exosomes bind preferentially to dendritic cells (DCs) for efficient antigen presentation.^21^ In addition to antigen presentation, exosomes serve as new mediators to promote the role of intestinal epithelial cells in maintaining intestinal mucosal homeostasis: thus maintaining the integrity of the epithelium, the production of mucus layers and the secretion of antibacterial peptides.^22^ Therefore, exosomes may be a part of IEC‐mediated innate immunity. They are also involved in immune regulation of Treg cells, known to play a role in maintaining immune tolerance and suppressing excessive immune response.^23^

Neddylation is a post‐translational protein modification in which a ubiquitin‐like protein NEDD8 (neural precursor cell‐expressed, developmentally downregulated gene 8) is covalently tagged to a targeted protein. In this process, NEDD8 is activated by NEDD8 enzyme E1 (heterodimer of NAe1 and Uba3), NEDD8 conjugated enzyme E2 (UBC12), and NEDD8 E3 ligase, after which it binds to a specific substrate protein, thereby forming a unique complex structure. NEDD8 combined with substrate does not degrade the protein, but rather regulates the conformation, stability, localization, and function of the substrate protein.^24^ The function of neddylation modification mainly reflects the following aspects^25^: (a) regulating the interaction between proteins; (b) regulating the activity of transcription factors to control the cell cycle and cell proliferation; and (c) antagonizing ubiquitination. Cullin neddylation leads to the activation of cullin‐RING ligase (CRL), which is the largest family of E3 ubiquitin ligases responsible for the ubiquitination and degradation of many key signaling or regulatory proteins. Neddylation regulates many biological processes by regulating CRLs, including cell cycle progression, signal transduction, and tumorigenesis. MLN4924 is a representative NEDD8‐activating enzyme (NAE) inhibitor, known to show a good inhibitory effect on NAE, thereby regulating the target protein function and expression level.^26^ By affecting the substrate protein neddylation, MLN4924 has potential effects on causing DNA damage, regulating the transmission of transcription factors, and inducing cancer cell apoptosis.^27^, ^28^

Current research has shown that neddylation plays an important regulatory role in IBD, but the mechanism by which human umbilical cord mesenchymal stem cell‐derived exosomes (hucMSC‐Ex) specifically regulate neddylation needs further exploration.

## 2 MATERIALS AND METHODS

The study was approved by the Ethical Committee of Jiangsu University (2012258).

### 2.1 Cell culture

MSCs were isolated and cultured in α‐MEM medium (Invitrogen) as previously described.^29^ Human colorectal mucosa cells (FHC) were purchased from Beiner Biotechnology and cultured in RPMI 1640 medium (Invitrogen). They were maintained in medium supplemented with 10% fetal bovine serum (FBS; Invitrogen) at 37°C in humidified air with 5% CO_2_.

### 2.2 Exosomal extraction

The MSCs were normally cultured in α‐MEM medium (containing 10% FBS). After 24 h, the supernatant was discarded, the cells were washed with PBS, followed by the addition of a new α‐MEM medium (10% without exosomal FBS). The supernatant was collected in sterile tubes after 48 h, and organelles were removed by centrifugation; during which cell debris was also removed. The resultant supernatant was poured into a 100 KD ultrafiltration centrifuge tube and repeatedly centrifuged until the supernatant was concentrated to 250 μL. Exosome extraction reagent (SBI) was added (supernatant:reagent = 5:1), and allowed to precipitate at 4°C overnight. The precipitated supernatant was centrifuged and washed to obtain hucMSC‐Ex. It was then resuspended in PBS, filtered through a 0.22 μm filter, and finally stored at −80°C.

### 2.3 NanoSight nanoparticle tracking analysis

HucMSC‐Ex was diluted with PBS, and 1 mL of suspension analyzed by NanoSight Nano Analyzer (Malvern).

### 2.4 TEM scanning

The diluted solution of hucMSC‐Ex in PBS was pipetted onto the copper net and made to stand at room temperature for 2‐3 min, after which it was counterstained with 3% phosphotungstic acid for 1‐2 min. The observation was done using an electron microscope (Philips).

### 2.5 Animal model establishment and treatment

Male BABL/C mice (6 weeks old, 20 g) were purchased from the Animal Research Center of Jiangsu University (Jiangsu, China), and related research was carried out after the approval by the Ethics Committee of Jiangsu University and the Experimental Animal Management and Use Committee.

Mice were randomly divided into three groups (n = 6/group): negative control group (Neg), DSS‐induced mouse IBD model group (DSS), and hucMSC‐Ex remission mouse IBD experimental group (hucMSC‐Ex). Mice in the Neg group drank autoclaved purified water, while both DSS group and hucMSC‐Ex group drank 3% DSS water in autoclaved purified water. In the hucMSC‐Ex group, 1 mg hucMSC‐Ex was injected into the tail vein of mice on the third, sixth, and ninth day after drinking 3% DSS, the other mice were injected with PBS. The body weights of the mice were recorded at the same time point every day, in addition to observation of the fecal characteristics and the presence of bloody stools. All the mice were sacrificed on the 10th day. The disease activity index (DAI) of the mice was analyzed (Table 3), and the tissues were also obtained for colorectal observation.

### 2.6 Animal live imaging analysis

1 mg hucMSC‐Ex and 1 μg DIR were incubated on a shaker at 37°C for 30 min. the hucMSC‐Ex‐DIR complex formed was collected by centrifugation and diluted in PBS. HucMSC‐Ex‐DIR was injected into the tail vein and 12 h later, a live imaging system was used to track the fluorescence distribution in the mice. The mice were sacrificed, and the colorectal tissue observed for fluorescence.

### 2.7 Co‐culture of FHC with hucMSC‐Ex

FHC concentration was adjusted to 1 × 10^6^/mL, followed by the addition of 2 mL cell suspension to each well of the six‐well plate. These cells were set up into negative control group (Neg), FHC + LPS group (LPS), FHC + LPS + hucMSC‐Ex group (hucMSC‐Ex), and FHC + LPS + MLN4924 group (MLN4924). 2 μg of LPS (100 ng/mL, Sigma), 200 μg of hucMSC‐Ex, and 0.2 μL of MLN4924 (MedChemExpress) were added to the respective groups. After co‐cultivation for 12 h, cells were collected for subsequent experiments.

### 2.8 Transfection

The transfection reagent (Gamma gene) was configured, 1 × 10^6^ FHC was added to each well of the six‐well plate. After 60% cellular adherence was obtained, the original culture solution was discarded, followed by the addition of blank 1640 without FBS (including transfection reagent). The cells were cultured in the dark for 6 h, after which the medium was replaced with a normal 1640 nutrient solution (containing 10% FBS) for an additional 48 h; the cells were then collected for subsequent analysis. HucMSC‐Ex was transfected with Exo‐Fect™ Exosome Transfection Reagent (SBI).

### 2.9 Hematoxylin and eosin (H&E) staining

A portion of the colorectal tissue (about 4 mm) was cut out and fixed in 4% paraformaldehyde. Following subsequent tissue processing, they were finally embedded in paraffin and sectioned with microtome. The sections were fixed on slides, dewaxed, and stained with H&E, followed by mounting and scanning using pathological section scanner.

### 2.10 Western blot analysis

RIPA lysate (Pierce) was added to the tissues/cells to obtain the protein that was mixed with the loading buffer (Life Technologies) at a ratio of 3:1, and boiled at 100°C for 8‐10 min. Protein samples were added to the gel well and the target protein was separated by electrophoresis. The protein was transferred to a PVDF membrane (Millipore), and non‐specific antigens blocked with 5% skim milk. The membranes were incubated with corresponding primary antibodies: anti‐CD9 (1:500; Proteintech), anti‐CD63 (1:500; Proteintech), anti‐CD81 (1:500; Proteintech), anti‐NEDD8 (1:500; CST), anti‐cullin 1 (1:500; Abcam), anti‐IκB (1:500; CST), anti‐NF‐κB (1:500; Abclonal), anti‐P‐NF‐κB (1:500; Abclonal), anti‐PCNA (1:500; Abcam), and anti‐β‐actin (1:500; SAB) overnight at 4°C. After washing with 1× TBS/T buffer, the membranes were incubated with the secondary antibody for 30 min at 37°C. Pictures were taken with a chemical gel imaging system (GE).

### 2.11 Coimmunoprecipitation

The Pierce TM Co‐Immunoprecipitation Kit (Thermo Fisher Scientific) was used to immobilize the antibodies in the resin, followed by Coimmunoprecipitation (Co‐IP). The eluted target protein was pretreated and then detected and analyzed by Western blot.

### 2.12 Real‐time fluorescence quantification PCR

Trizol (Gibco) was added to the tissues/cells, and RNA was obtained by chloroform extraction. cDNA was obtained through a corresponding reverse transcription kit (Vazyme), real‐time fluorescence quantification PCR (qRT‐PCR) was carried out in a Step One Plus Real‐Time PCR System (ABI) to detect gene expression. The sequences of primers used are listed in Tables 1 and 2.

**TABLE 1:** Primer sequence

Species	Gene	Primer sequence	Temperature
Mouse	β‐actin	FOR: CTCAGGAGGAGCAATGATCT	58°C
		REV: GACCTGTACGCCAACACAGT	
	IL‐1β	FOR: AGCTTCAGGCAGGCAGTATC	61°C
		REV: TCATCTCGGAGCCTGTAGTG	
	IL‐6	FOR: AAGTCCGGAGAGGAGACTTC	58°C
		REV: TGGATGGTCTTGGTCCTTAG	
	IL‐10	FOR: CCTGGCTCAGCACTGCTATG	58°C
		REV: TCACCTGGCTGAAGGCAGTC	
	TNF‐α	FOR: AACTCCAGGCGGTGCCTATG	63°C
		REV: TCCAGCTGCTCCTCCACTTG	
	NAe1	FOR: GCAACGGCTACAGGAACTGA	60°C
		REV: GCTCGGTTCTTGCCAATACT	
	Uba3	FOR: GCTGGTGGCTTAGGATGTGA	60°C
		REV: GTACCACGTTGCAGTTAG	
	UBC12F	FOR: CCAAGGGCAGCAGCAAGA	58°C
		REV: AGTGGGTCCTCAGGGTTCG	
	DCNL1	FOR: GGGGTTCAGTCTTCGTGT	58°C
		REV: TTATTGCCTCCGTGGGTA	
	E2M	FOR: AGACGACCTCCTCAACTTCA	58°C
		REV: TCGGCTCCAAGAAGAGATAC	

**TABLE 2:** Primer sequence

Species	Gene	Primer sequence	Temperature
Human	NAe1	FOR: AATGTTACGGGCTGTTGA	60°C
		REV: AAGGTGCTTGCTTACTCTAC	
	Uba3	FOR: TTGCCACGATTTGTCTTT	60°C
		REV: CTCCAGGTATCATTTCTCAT	
	UBC12F	FOR: AGCTGTTCTCGCTGAAGCA	58°C
		REV: AAGTTGAGGAGGTCGTCTGG	

**TABLE 3:** The clinic disease activity index (DAI) value

Values	Weight loss (%)	Stool consistency	Rectal bleeding
0	None	Normal	Normal
1	1‐5		±
2	5‐10	loose stool	+
3	10‐15		++
4	more than 15	watery diarrhea	+++

DAI = (Weight loss + Stool consistency + Rectal bleeding)/3.

### 2.13 Immunohistochemistry (IHC)

The tissue embedded in paraffin were fixed and dewaxed, followed by endogenous peroxidase blocking with 3% hydrogen peroxide solution at room temperature for 30 min. Tissues were steamed for 30 min in citrate buffer to repair antigens and incubated with 5% BSA solution for 30 min to block non‐specific antigens. The tissues were then incubated with antibodies: anti‐NEDD8 (1:200; CST), and anti‐cullin 1 (1:200; Abcam) at 4°C for overnight, and with the secondary antibody at 37°C for 30 min. Subsequently, tissue color rendering by DAB (Biorad) was used, resin sealing applied, and pathological section scanner used to obtain pictures.

### 2.14 Immunofluorescence (IF)

Cells obtained from culture were washed with PBS and fixed in 4% paraformaldehyde at room temperature for 30 min. Triton X‐100 (0.1%) was added, gently mixed for 30 min, and non‐specific antigens blocked by the addition of 5% BSA solution. The cells were incubated with the antibodies: anti‐NEDD8 (1:200; CST), and anti‐cullin 1 (1:200; Abcam) overnight at 4°C, then incubated with fluorescent secondary antibody at 37°C for 60 min. They were finally incubated with honchest (Biorad) for 10 min at room temperature, and anti‐quenching agent, after which pictures were taken by scanning with a confocal laser microscope (Nikon). The light was avoided in the entire process.

### 2.15 CCK8

A total of 100 μL of FHC suspension was added to a 96‐well plate. When the cell density reached 60%, the nutrient solution was replaced and CCK8 solution (Vazyme) added at 12, 24, 36, and 48 h to the culture wells. The resultant cell suspensions were incubated for 30 min in the dark, and absorbance measured at 450 nm with microplate reader (Thermo Fisher Scientific).

### 2.16 Luciferase reporter assay of miRNA target

The NEDD8 mRNA 3′‐UTR region containing the miR‐326 binding site, WT/Mut (GGCCCAGAG mutated to gcaagcggc), was cloned into a dual‐luciferase miRNA target expression vector (GP‐miRGLO, Gamma gene). The two vectors were co‐transfected into FHC with miR‐326 mimics, mimics NC, inhibitor, and inhibitor NC, and then cultured for 12 h. Dual‐luciferase reporter gene kit (Promega) was used to detect related expression.

### 2.17 Image and statistical analysis

All data were shown as mean ± SEM. Statistical analysis was performed by using GraphPad Prism software for analysis of variance (ANOVA), with *P* < .05 considered statistically significant.

## 3 RESULTS

### 3.1 Extraction and identification of hucMSC‐Ex

**FIGURE 1:**
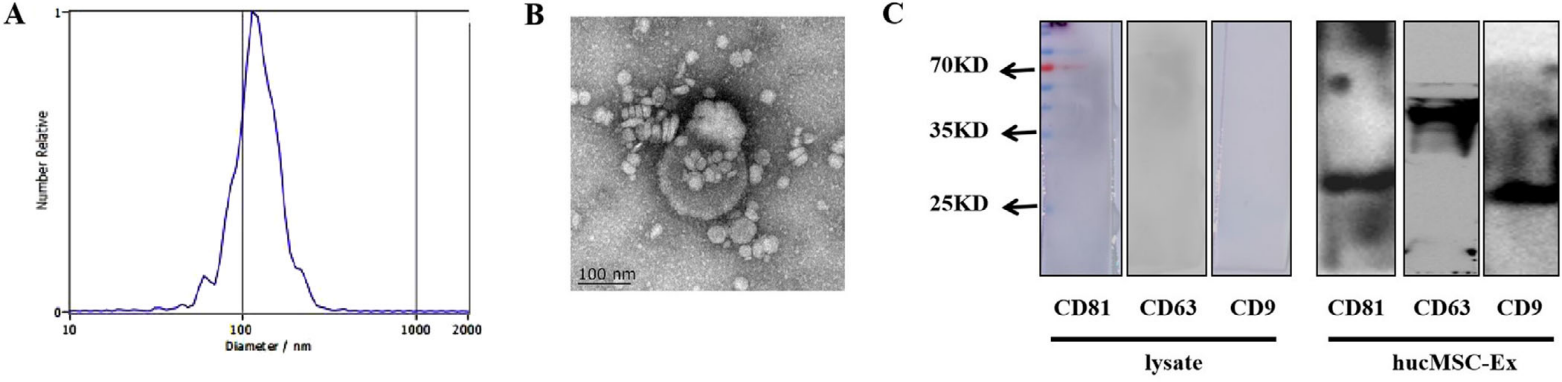
Identification of hucMSC‐Ex. A, NanoSight Nanoparticle Tracking Analyzer detection of hucMSC‐Ex diameters; B, Transmission electron microscope detection of hucMSC‐Ex separation and purification; C, Western blot analysis of hucMSC‐Ex related surface markers

The results of NanoSight and TEM showed that the extracted hucMSC‐Ex was 30‐200 nm in diameter, vesicle‐like, and highly concentrated (Figure 1A,B; Figure S1). At the same time, western blot results showed that hucMSC‐Ex expressed CD9, CD63, and CD81 (Figure 1C).

### 3.2 HucMSC‐Ex relieves DSS‐induced mouse IBD

**FIGURE 2:**
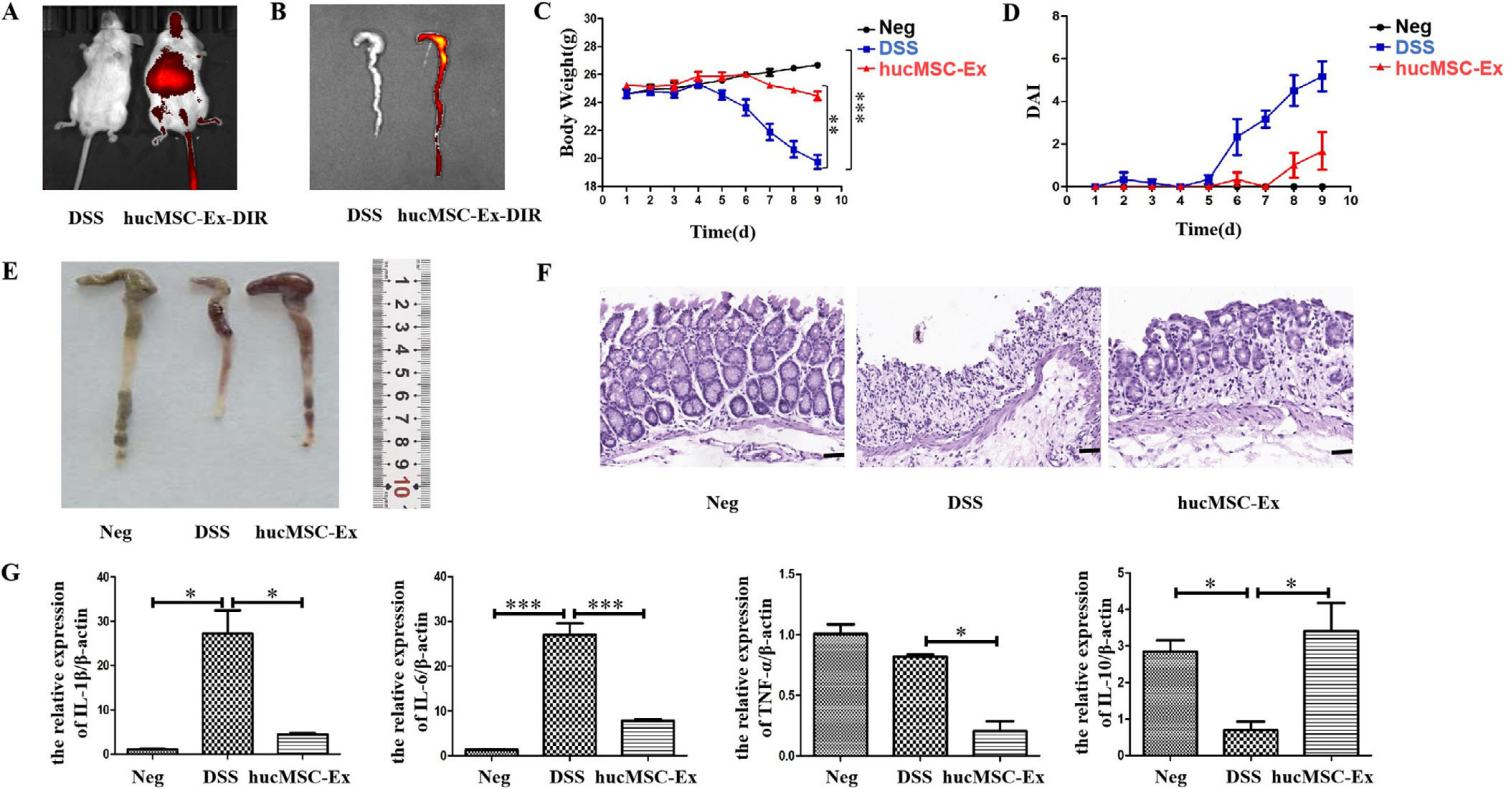
HucMSC‐Ex relieves DSS‐induced mouse IBD. A, Fluorescence distribution of DIR‐labeled hucMSC‐Ex in mouse; B, Fluorescence distribution of DIR‐labeled hucMSC‐Ex in mouse colon tissue; C, Changes in mice weight; D, DAI scores of mice; E, Colon appearance in mice; F, H&E staining of mice colon tissue (scale bar = 50 μm); G, QRT‐PCR analysis of inflammatory factors expression in mice colon tissue, ^*^ *P* < .05; ^**^ *P* < .01; ^***^ *P* < .001 by ANOVA

To determine the effects of hucMSC‐Ex in damaged colon tissue, we labeled hucMSC‐Ex with DIR fluorescent dye and injected it into IBD mice. 12 h post‐injection, treated mice (with hucMSC‐Ex‐DIR) showed a large area of fluorescence in vivo (Figure 2A). The mice were subsequently sacrificed, and the colorectal tissue further analyzed. Colon tissue of hucMSC‐Ex‐DIR‐treated IBD mice expressed strong fluorescence, but the colorectal tissue of untreated group did not express fluorescence (Figure 2B).

The weight of the mice in the Neg group increased with time, but the weight of the mice in the DSS group decreased on the fifth day with blood also appearing in stool. The body weight of the mice in the hucMSC‐Ex group basically remained unchanged in the first 6 days, but slightly decreased after day 6 though not obvious (Figure 2C). Although the DAI of all the groups did not increase in the first 5 days, that of the DSS group increased sharply on the sixth day, and continued the upward trend. The DAI of the hucMSC‐Ex group also began to increase on the sixth day, but turned a downward trend from the seventh day (Figure 2D). All mice were sacrificed, and their colon tissues were taken for further observations. While the colon tissue of mice in the Neg group was longer (87 mm), that of the DSS group was significantly shorter (64 mm). After treatment with hucMSC‐Ex, the colon length recovered (77 mm) (Figure 2E). H&E staining results showed that DSS induced intestinal inflammation led to severe villi structural damage, but treatment with hucMSC‐Ex restored the structural integrity of the colon tissue (Figure 2F). QRT‐PCR results showed that compared to mice in the Neg group, pro‐inflammatory factors (IL‐1β, IL‐6) were increased in colon tissue of the DSS group, but significantly decreased in the hucMSC‐Ex group. Conversely, the expression of anti‐inflammatory factor (IL‐10) was reduced in the colon tissue of the DSS group, while increased in the hucMSC‐Ex group (Figure 2G).

### 3.3 HucMSC‐Ex inhibits neddylation to relieve IBD

**FIGURE 3:**
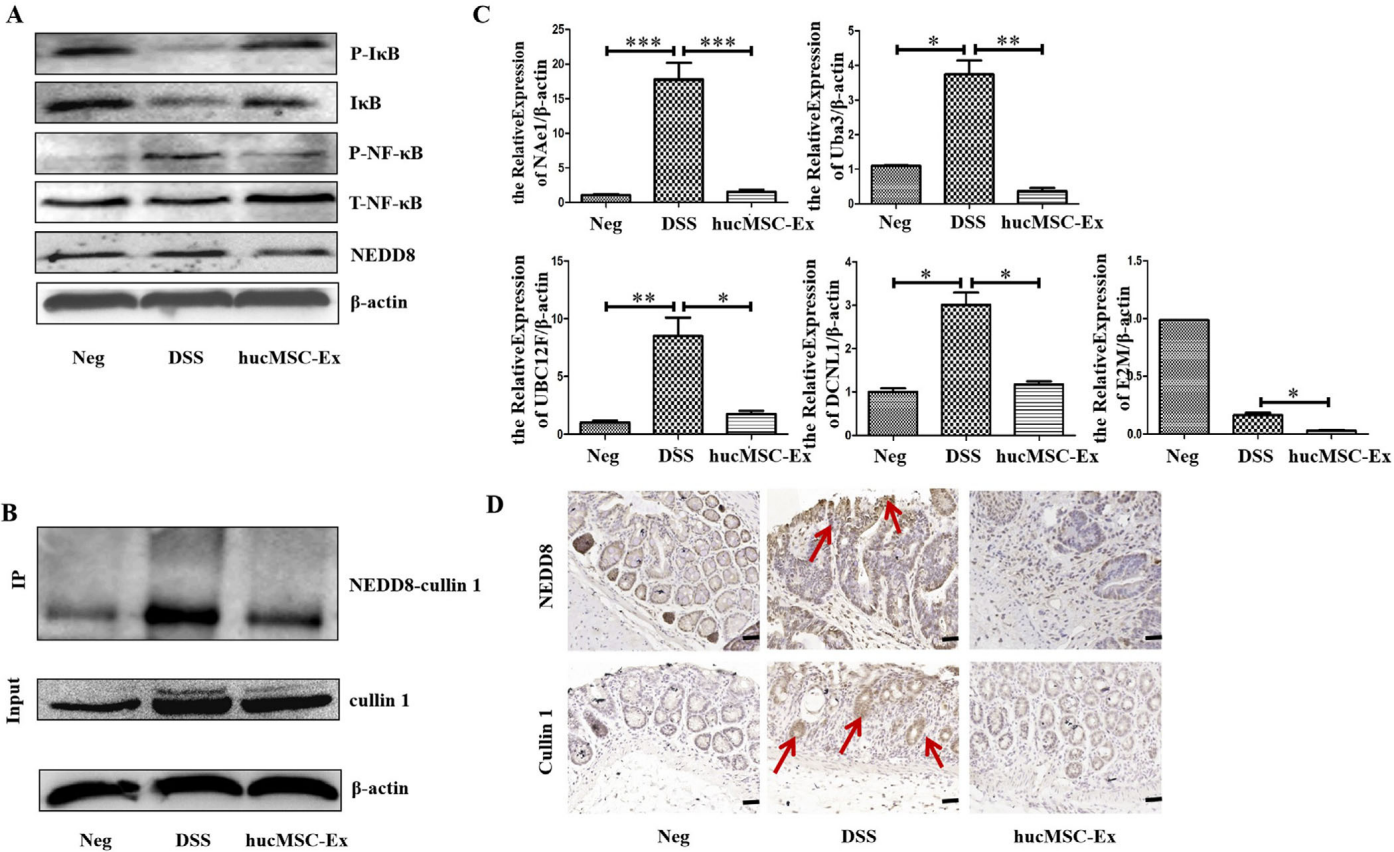
HucMSC‐Ex suppresses neddylation process to relieve IBD. A, Western blot analysis of the expression level of neddylation‐related molecules in colon tissue; B, Co‐IP analysis of the binding of NEDD8 to cullin 1; C, QRT‐PCR analysis of the expression level of neddylation‐related molecules in colon tissue, ^*^ *P* < .05; ^**^ *P* < .01; ^***^ *P* < .001 by ANOVA; D, IHC analysis of colon tissue level of neddylation‐related molecules (scale bar = 50 μm)

Western blot results showed that NEDD8 was overexpressed in the colon tissue of DSS mice, and IκB expression was decreased. However, NEDD8 decreased in hucMSC‐Ex treated mice with inhibited IκB degradative activity. At the same time, P‐NF‐κB expression was upregulated in the DSS group and decreased in the hucMSC‐Ex group (Figure 3A; Figure S2). Co‐IP results showed an enhanced binding of NEDD8 to cullin 1 in the colon tissue of the DSS group, but hucMSC‐Ex treatment inhibited this activity (Figure 3B). In addition to the binding of NEDD8 to cullin 1, qRT‐PCR results also showed significantly increased expression of neddylation‐related molecules (NAe1, UBA3, UBC12F, DCNL1) in the colon tissue of the DSS group, but decreased after treatment with hucMSC‐Ex (Figure 3C), the expression levels were statistically significant (*P* < .05). For a more intuitive observation of the expression of NEDD8 and cullin1 in the colon tissue, IHC was performed, and results showed increased levels of NEDD8 and cullin1 in DSS group but decreased levels in hucMSC‐Ex treated group (Figure 3D).

### 3.4 HucMSC‐Ex inhibits neddylation process of FHC in an inflammatory environment

**FIGURE 4:**
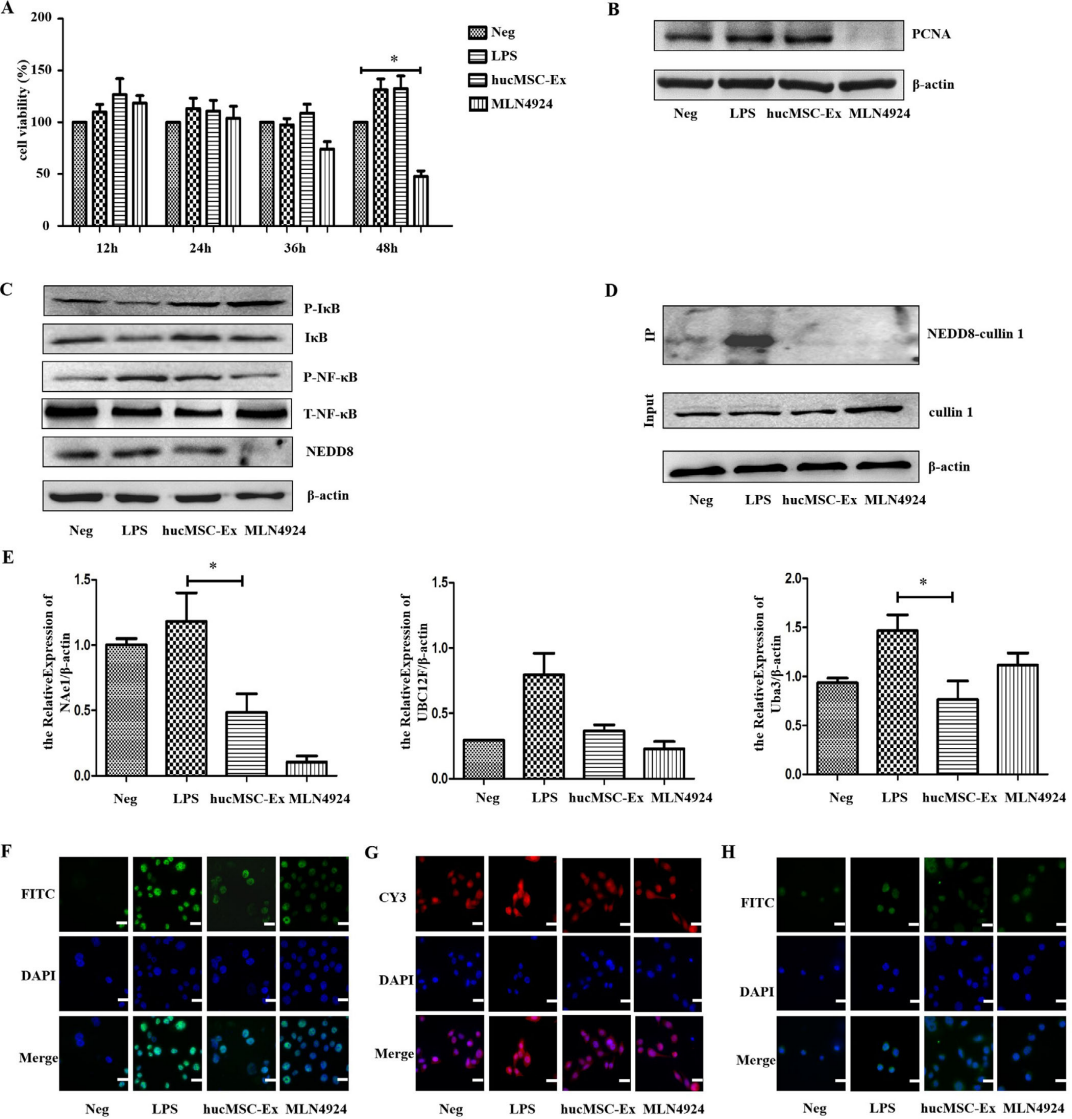
*HucMSC‐Ex inhibits neddylation process of FHC in inflammatory environment* (A) CCK8 analysis of FHC proliferation, *P < 0.05; **P < 0.01; ***P < 0.001 by ANOVA; (B) Western blot analysis of FHC proliferation; (C) Western blot analysis of neddylation‐related molecules in FHC; (D) Co‐IP analysis of binding of NEDD8 to cullin 1; (E) QRT‐PCR analysis of neddylation‐related molecules expression in FHC, *P < 0.05; **P < 0.01; ***P < 0.001 by ANOVA ; (F) IF analysis of expression of neddylation‐related molecules in FHC (cullin 1) (scale bar = 50 μm); (G) IF analysis of neddylation‐related molecules in FHC (NEDD8) (scale bar = 50 μm); (H) IF analysis of P‐NF‐κB in FHC (scale bar = 50 μm)

Cell counting kit‐8 (CCK8) experiment indicated that cell proliferation did not change after 12, 24, 36, and 48 h when FHC was induced by LPS. However, after co‐culture with MLN4924, although the cell proliferation did not change at 12 and 24 h, the cell viability began to decline after 36 h, and at 48 h, viability had significantly decreased (Figure 4A), *P* < .05. Western blot showed that after 12 h of cell culture, the Neg, LPS, and hucMSC‐Ex groups all expressed proliferating cell nuclear antigen (PCNA), but the expression level in cells of LPS and hucMSC‐Ex groups increased. On the other hand, PCNA expression in cells of MLN4924 group significantly decreased (Figure 4B; Figure S3A).

From the background that hucMSC‐Ex and MLN4924 had no effect on FHC proliferation at 12 h, few other investigations were performed. Western blot analysis showed overexpression of NEDD8 but decreased IκB in FHC of the LPS group. Again, NEDD8 expression was decreased in the MLN4924 group cells, and IκB degradation was inhibited. At the same time, P‐NF‐κB expression was upregulated in the LPS group and decreased in the MLN4924 group. The expression changes of NEDD8, IκB, and P‐NF‐κB in cells of HucMSC‐Ex group were consistent with those of MLN4924 group (Figure 4C; Figure S3B,C). Co‐IP results showed that although NEDD8 binds to cullin 1 in the control, the degree of binding in the LPS group is significantly enhanced, but inhibited in hucMSC‐Ex and MLN4924 groups (Figure 4D). QRT‐PCR analysis indicated significantly increased cellular expression of neddylation‐related molecules (NAe1, UBC12F, UBA3) in the LPS group, but decreased expression in hucMSC‐Ex and MLN4924 groups (Figure 4E). IF analysis of FHC showed that the fluorescence intensity of cullin1 in the cells of LPS group was significantly enhanced and mainly concentrated in the nucleus, while hucMSC‐Ex and MLN4924 groups decreased (Figure 4F). Similarly, the fluorescence intensity of NEDD8 in FHC of LPS group was significantly enhanced with stronger expression in both the cytoplasm and nucleus, while that of hucMSC‐Ex and MLN4924 groups decreased (Figure 4G). The overall fluorescence intensity of P‐NF‐κB in the hucMSC‐Ex and MLN4924 groups decreased, thus less fluorescence in the nucleus and cytoplasm (Figure 4H).

### 3.5 HucMSC‐Ex‐expressed miR‐326 inhibits neddylation

**FIGURE 5:**
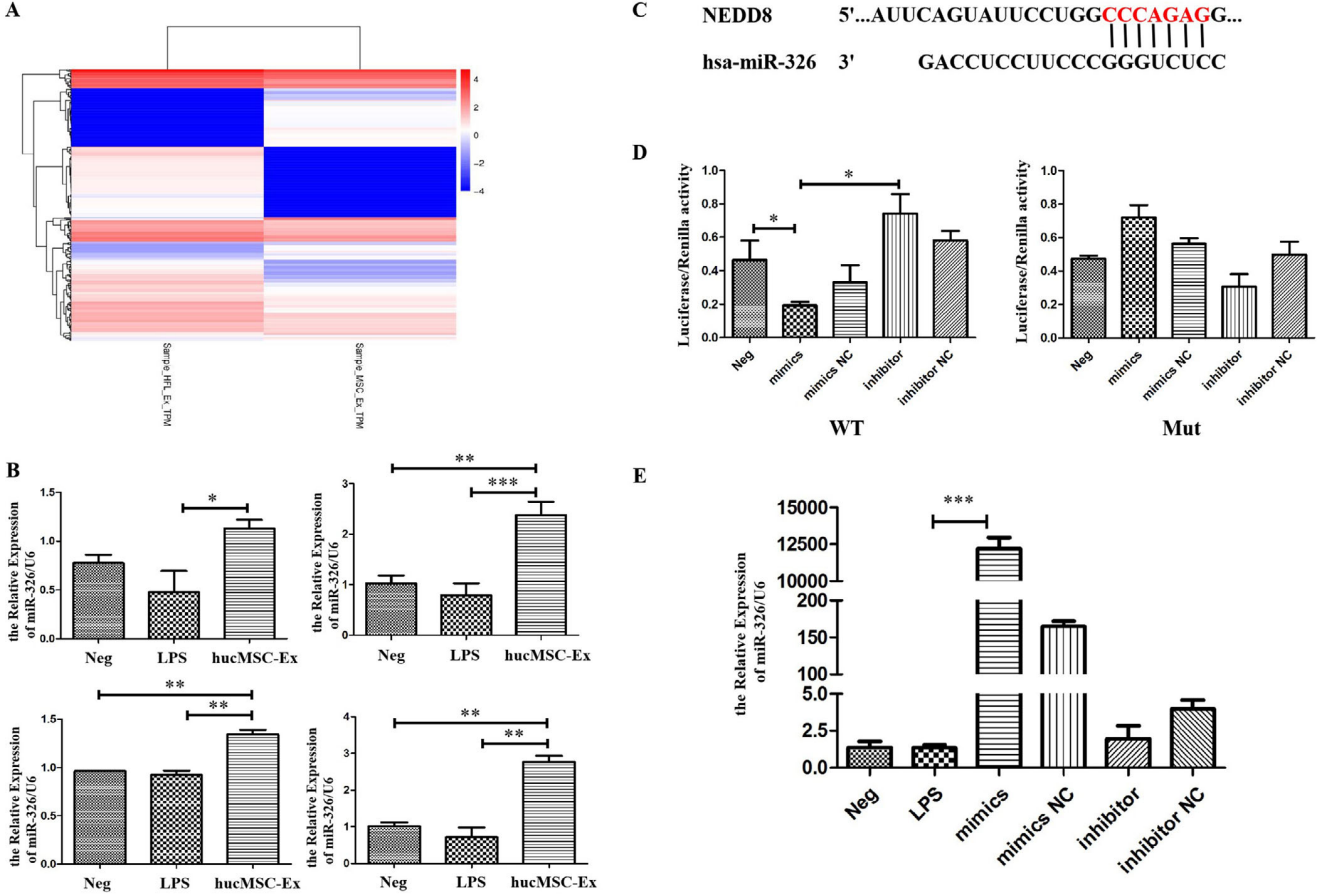
miR‐326 as key molecule in hucMSC‐Ex. A, Sequencing results of miRNAs in hucMSC‐Ex and HFL1‐Ex; B, QRT‐PCR analysis of miR‐326 expression levels in FHC treated with hucMSC‐Ex; C, Binding sites between NEDD8 and miR‐326; D, Dual‐luciferase reporter gene of the targeting relationship between miR‐326 and NEDD8; E, QRT‐PCR analysis of miR‐326 expression levels in FHC after transfection. ^*^ *P* < .05; ^**^ *P* < .01; ^***^ *P* < .001 by ANOVA

Both in vivo and in vitro experiments have shown that hucMSC‐Ex can inhibit the process of neddylation, but key molecules within hucMSC‐Ex involved in this regulatory process need further study. We used Illumina Hiseq (Oebiotech, OE2015H1459) to sequence hucMSC‐Ex and HFL1‐Ex separately for comparison (Figure 5A, source: Key Laboratory of Medical Science and Laboratory Medicine of Jiangsu Province, School of Medicine, Jiangsu University), and made prediction of possible miRNAs that could target NEDD8 using http://www.targetscan.org website. Alignment results singled out four miRNAs: miR‐27b, miR‐326, miR‐411‐5p, and miR‐665. To verify the cellular expression of selected miRNAs, FHC was treated with different batches of hucMSC‐Ex. QRT‐PCR results showed that only miR‐326 was stably and significantly expressed in cells under hucMSC‐Ex treatment, and less expressed in Neg and LPS groups (Figure 5B). Based on this outcome, the probable binding site between miR‐326 and NEDD8 mRNA 3′URT was predicted (Figure 5C). The result showed that the ratio of the two luciferase activities of the WT in the miR‐326 mimics group was significantly reduced. In contrast, the activity ratio of the inhibitor group increased, which was statistically significant (*P* < .05). There was no significant difference in the ratio of luciferase activity (Figure 5D). FHC was transfected with miR‐326 mimics, mimics NC, inhibitor, and inhibitor NC for 48 h. QRT‐PCR results showed that miR‐326 was significantly overexpressed in miR‐326 mimics cells but no significant change in miR‐326 inhibitor group (Figure 5E).

**FIGURE 6:**
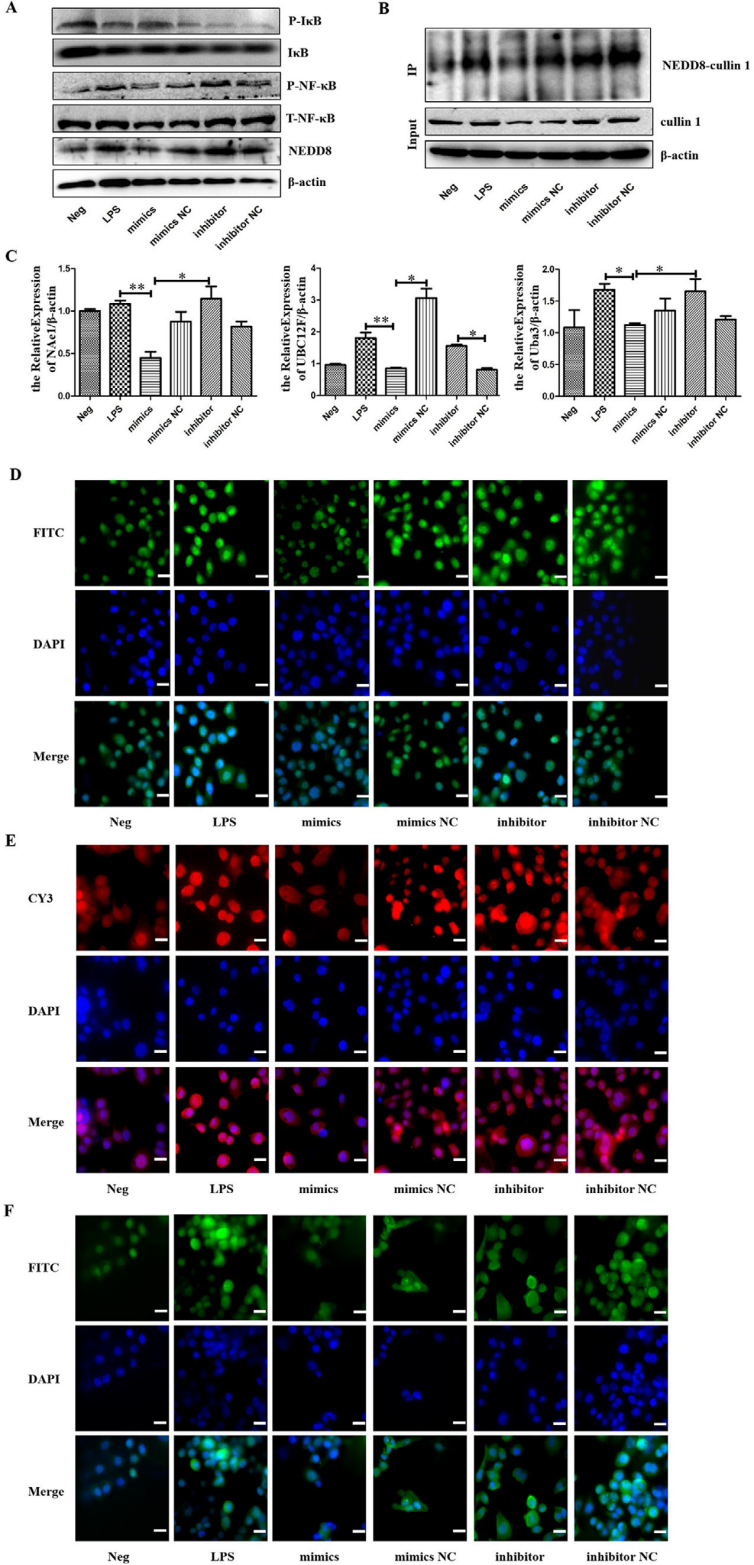
HucMSC‐Ex expressing miR‐326 inhibits neddylation. A, Western blot analysis of neddylation‐related molecules in FHC; B, Co‐IP analysis of binding of NEDD8 to cullin 1; C, QRT‐PCR analysis of neddylation‐related molecules expression in FHC, ^*^ *P* < .05; ^**^ *P* < .01; ^***^ *P* < .001 by ANOVA; D, IF analysis of neddylation‐related molecules in FHC (cullin 1) (scale bar = 50 μm); E, IF analysis of neddylation‐related molecules in FHC (NEDD8) (scale bar = 50 μm); F, IF analysis of P‐NF‐κB expression in FHC (scale bar = 50 μm)

FHC was successfully transfected with miR‐326 mimics and miR‐326 inhibitor for further studies on the regulation of neddylation. Western blot results showed that NEDD8 and P‐NF‐κB were overexpressed in FHC of the LPS group, but IκB expression showed a slight reduction. On the other hand, NEDD8 and P‐NF‐κB decreased in mimics group, while IκB degradation was slightly inhibited. In contrast to the LPS group, the expression of NEDD8 and P‐NF‐κB in the inhibitor group was enhanced, while IκB was further reduced, but no significant change in the mimics NC and inhibitor NC groups (Figure 6A; Figure S4). Co‐IP results showed that the binding degree of NEDD8 to cullin 1 in LPS and inhibitor groups was significantly enhanced, but inhibited in mimics group (Figure 6B). Upon qRT‐PCR analysis, there was a significant expression of neddylation‐related molecules (NAe1, UBC12F, Uba3) in the LPS group, but the reduced expression in mimics group (Figure 6C). IF results also showed that while the fluorescence intensity of cullin 1 in the cells of the LPS group was significantly enhanced but reduced in the mimics group, the fluorescence in the inhibitor group had no significant reduction (Figure 6D). The expression of NEDD8 followed a similar trend (Figure 6E). Again, the fluorescence of P‐NF‐κB in the LPS group was enhanced and mainly concentrated in the nucleus. The overall fluorescence of miR‐326 mimics group cells was significantly weak and reduced in the nucleus but appeared in the cytoplasm, while that of miR‐326 inhibitor group had no significant reduction in the nucleus (Figure 6F).

### 3.6 HucMSC‐Ex miR‐326 relieves IBD in mice by inhibiting neddylation

**FIGURE 7:**
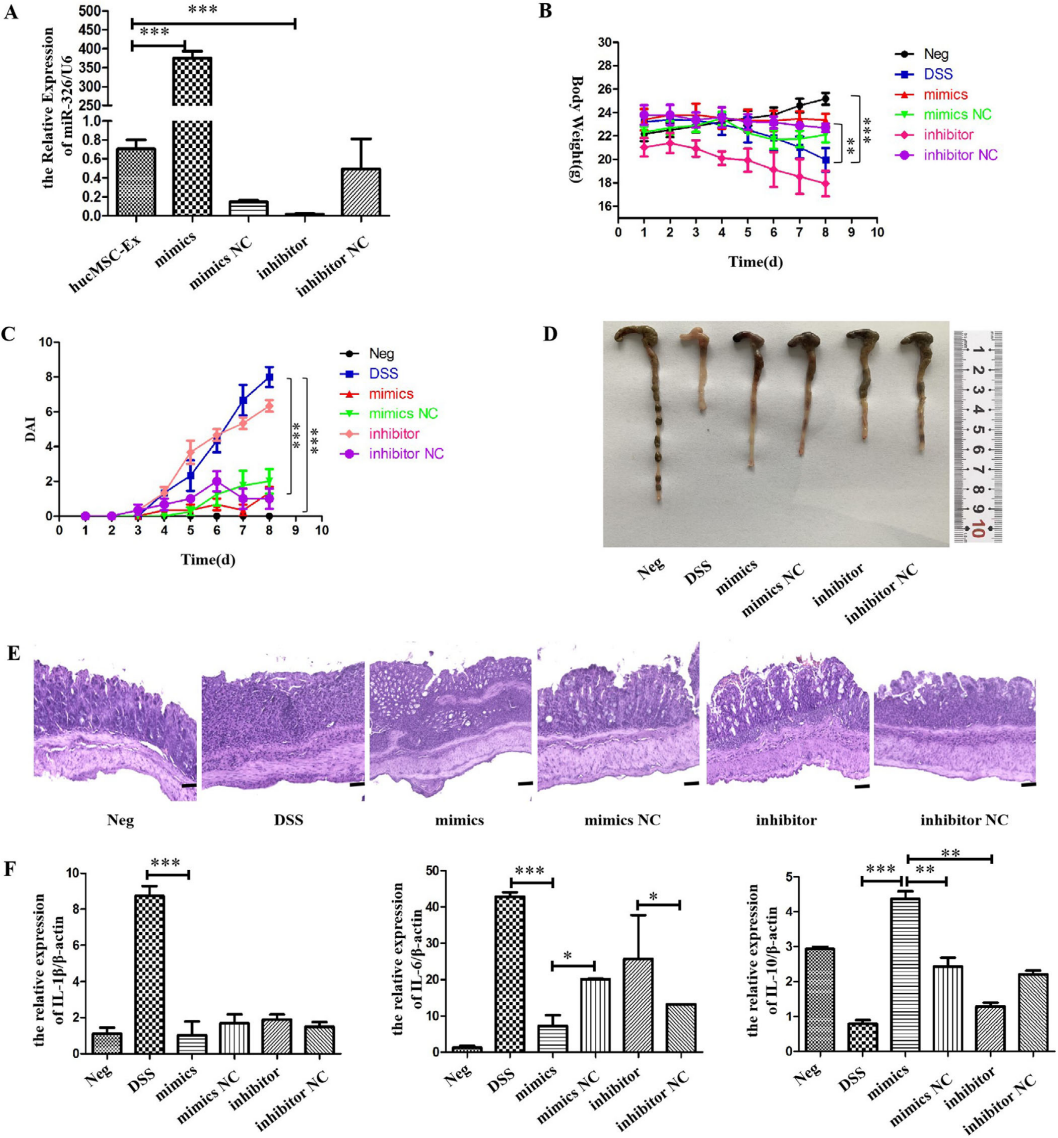
HucMSC‐Ex overexpressing miR‐326 relieves DSS‐induced IBD. A, QRT‐PCR analysis of miR‐326 expression level in hucMSC‐Ex after transfection; B, Changes in mice weight; C, DAI scores of mice; D, Colon appearance of mice; E, H&E staining of mouse colon tissue (scale bar = 50 μm); F, QRT‐PCR analysis of inflammatory factors expressed in mouse colon tissue, ^*^ *P* < 0.05; ^**^ *P* < 0.01; ^***^ *P* < 0.001 by ANOVA

In order to verify the effect of miR‐326 in IBD mice, hucMSC‐Ex was transfected with miR‐326 mimics, mimics NC, inhibitor, and inhibitor NC, for subsequent treatment of IBD. The initial qRT‐PCR analysis showed that miR‐326 expression was significantly upregulated in the mimics group but reduced in the inhibitor group (Figure 7A) indicating a successfully constructed miR‐326 overexpressed/underexpressed hucMSC‐Ex for subsequent experiments.

The trend of weight loss in the mimics group was suppressed compared to mimics NC group, with reduced DAI score. Similarly, mice in the inhibitor group had a more significant weight loss and higher DAI score than those of inhibitor NC group (Figure 7B,C). After treatment, all mice were sacrificed and colon taken for further observation. The colon of mimics group (75 mm) was significantly longer than those of DSS (42 mm) and mimics NC groups (65 mm). The mice of the inhibitor group (57 mm) had shorter colon than inhibitor NC group (63 mm), but longer than the DSS group (42 mm; Figure 7D). H&E staining results showed that the intestinal villous structure of DSS group was severely damaged, while that of mimics group was restored. The structural integrity of the colonic tissue of mimics NC group was partially recovered, and that of the inhibitor group was relatively disordered (Figure 7E). QRT‐PCR results showed the reduced expression level of pro‐inflammatory factors (IL‐1β, IL‐6) in mimics group compared to mimics NC group. Similarly, the expression was lower in the inhibitor NC group than the inhibitor group (Figure 7F).

**FIGURE 8:**
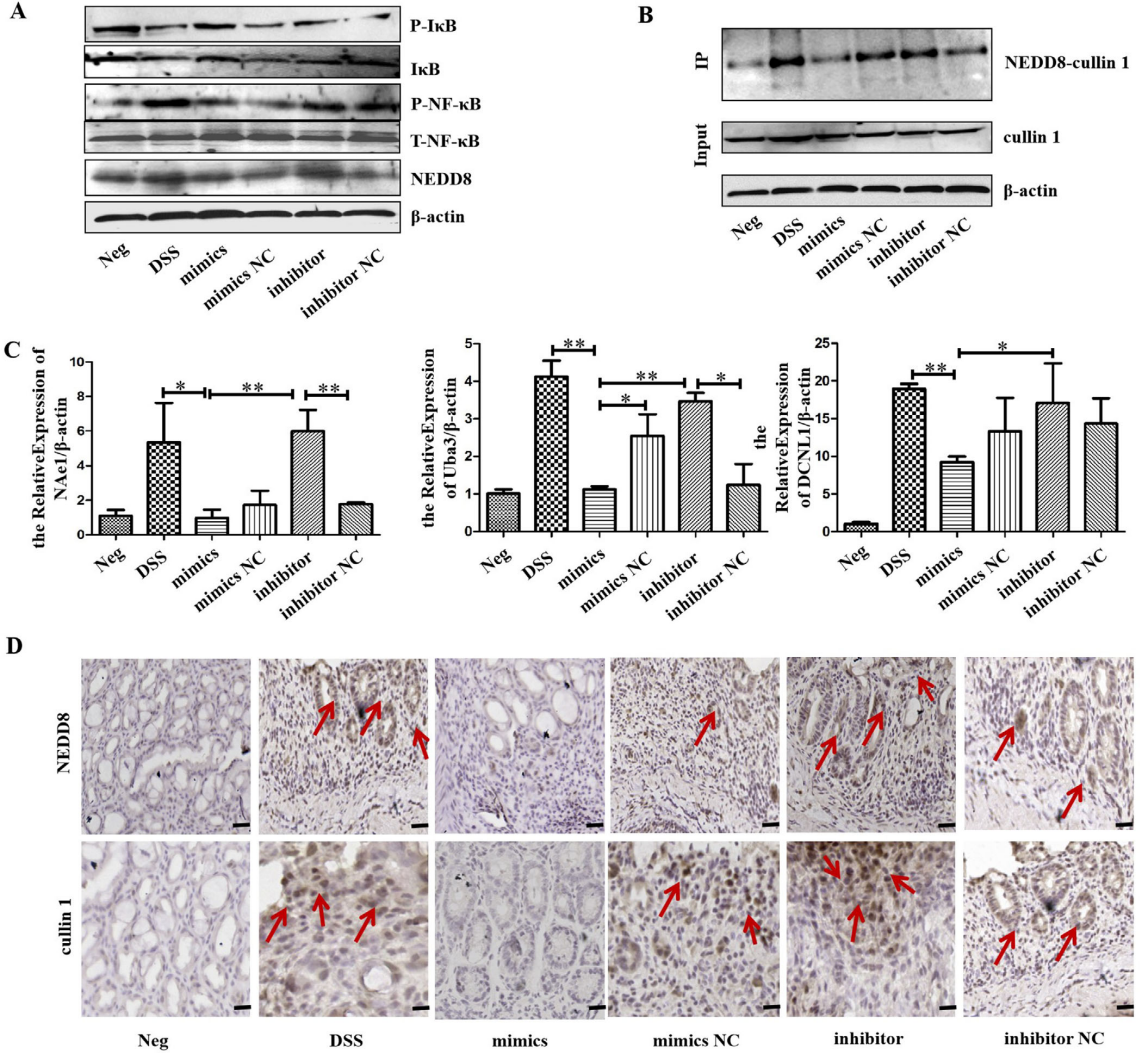
HucMSC‐Ex overexpressing miR‐326 suppresses neddylation process during to relieve IBD. A, Western blot analysis of the expression level of neddylation‐related molecules in colon tissue; B, Co‐IP analysis of the binding of NEDD8 to cullin 1; C, QRT‐PCR analysis of the expression level of neddylation‐related molecules in colon tissue, ^*^ *P* < .05; ^**^ *P* < .01; ^***^ *P* < .001 by ANOVA; D, IHC analysis of colon tissue level of neddylation‐related molecules (scale bar = 50 μm)

Western blot analysis indicated reduced expression of NEDD8 but increased expression of IκB in mimics group compared to mimics NC group, and higher expression of NEDD8 but promoted degradation of IκB in inhibitor group. P‐NF‐κB was down‐regulated in the mimics group and up‐regulated in the inhibitor group (Figure 8A; Figure S5). Co‐IP results showed that the degree of binding of NEDD8 to cullin 1 was inhibited in the mimics group and enhanced in the inhibitor group (Figure 8B). QRT‐PCR showed reduced expression of neddylation‐related molecules (NAe1, Uba3, DCNL1) in the colon tissues of mice in the mimics group and increased in the inhibitor group (Figure 8C). Analysis by IHC showed weak expression of NEDD8 and cullin 1 in the mimics group. Compared to the inhibitor NC group, the NEDD8 and cullin 1 in the colon tissue of the inhibitor group increased (Figure 8D).

## 4 DISCUSSION

Common proteins in exosomes are members of the tetraspanin (TM4SF) superfamily. The TM4SF family is a group of scaffold membrane proteins including CD63, CD81, and CD9. They are located on the surface of exosomes and serve as detection markers.^30^ Exosomes also contain messenger RNA (mRNA) and miRNA. When exosomes are endocytosed by recipient cells, the genetic information carried by RNA affects protein expression in these cells.^31^, ^32^ Exosomes can be used for disease diagnosis, drug delivery, and as therapeutic agents.^33^ Because exosomes contain unique biologically active molecules, they represent the composition, physiological state, and characterization of parent cells,^34^ hence they can also be referred to as the “fingerprints” of parent cells.^35^ The structure of exosomes is similar to liposomes, so it can protect its contents from the external environment, thus maintaining the integrity of its biological activity. Exosomes can mediate cell‐to‐cell communication between various parts of the human body, and are transported in the body through the blood yet avoid inducing immune responses.^36^ Compared with the uncontrolled growth of cells in cell therapy, exosomes will not mutate, replicate, or induce metastasis, making them safer and easier to control. Collectively, these functions support exosomes making them potential therapeutics and delivery tools. In recent years, some studies have shown that intestinal epithelial‐derived exosomes repair intestinal mucosal damage through ANXA1,^37^ and exosomes secreted by bone marrow‐derived DC can relieve 2,4,6‐trinitrobenzenesulfonic acid (TNBS)‐induced colitis.^38^ *Schistosoma japonicum* and hookworm‐derived exosomes alleviate intestinal mucosal damage by inhibiting the secretion of pro‐inflammatory factors.^39^, ^40^ Currently, stem/progenitor cells, especially MSCs, are important active ingredients in regenerative medicine.^41^ This study shows that after injecting hucMSC‐Ex through the tail vein into IBD mice, the hucMSC‐Ex can reach the damaged colon tissue and act on the target tissue through direct contact or paracrine mode to exert its effect. HucMSC‐Ex treatment mitigated clinical symptoms associated with IBD (weight loss, shortened colon, and bloody stool), restored structural integrity of colon tissue, and inhibited secretion of pro‐inflammatory factors in colon tissue.

NF‐κB is a ubiquitous pro‐inflammatory transcription factor in mammalian cells and plays a key role in regulating the production of pro‐inflammatory factors (IL‐1β, TNF‐α).^42^, ^43^ IκB kinase complexes are part of the upstream NF‐κB signal transduction cascade. IκB proteins inactivate NF‐κB transcription factors by masking the nuclear localization signals of NF‐κB proteins to keep them inactive in the cytoplasm. It has been shown that the deterioration of IκB is mainly ubiquitination, and that this process is dependent on neddylation. When cullin activation is blocked, it leads to accumulation of many CRL substrates including IκB, which inhibits NF‐κB activity.^44^, ^45^ Cullin 1 is one of the most important substrates in neddylation, and its expression level can indirectly show the progress of neddylation. The intensity of neddylation depends on the expression level of free NEDDB. Free NEDDB is activated by E1 activase and transferred to E2 ligase, and then to E3 ligase to get it activated.^46^ NAE is currently the only E1 activating enzyme (dimer formed by NAe1 and Uba3), and UBC12F is an E2 ligase involved in the neddylation modification process. Five DCNL (DCN1‐like proteins) can be found in mammals, and DCNL1 plays a role in cullin 1 neddylation as E3 ligase.^47^, ^48^ In this study, we have demonstrated that in the process of relieving IBD in mice, hucMSC‐Ex inhibits the expression level of free NEDD8 and thus prevents the binding of NEDD8 to cullin 1. Related enzymes involved in the neddylation process including E1 activases (NAe1, Uba3), E2 ligase (UBC12F), and E3 ligase (DCNL1), also had reduced expression levels, indicating the inhibitory effect of hucMSC‐Ex on neddylation. Additionally, IκB (a substrate of CRL), was inhibited by hucMSC‐Ex, and its degradation effect alleviated. IκB accumulation caused inhibition of NF‐κB phosphorylation.

As a selective NAE inhibitor, MLN4924 regulates multiple signaling pathways by inhibiting the neddylation of target molecules.^49^ FHC is a human colorectal mucosal cell that grows steadily in nutrient solutions. This study demonstrated that LPS and hucMSC‐Ex had no significant effect on the growth of FHC within 48 h, but the inhibitory effect of MLN4924 on cell proliferation became more obvious as time progressed. Although MLN4924 is known to effectively inhibit neddylation, long‐term damage to cells cannot be ignored, and it is currently used clinically as a tumor suppressor.^49^ This study showed that treatment with hucMSC‐Ex after LPS‐induced acute inflammation of FHC, resulted in similar inhibitory effect to MLN4924. For example, both of them inhibited the expression of NEDD8 and thus prevented the binding of NEDD8 to cullin 1. The expression levels of related enzymes were reduced, IκB accumulated, and NF‐κB phosphorylation was inhibited. At the same time, the transfer of P‐NF‐κB from the cytoplasm to the nucleus was inhibited. These results indicate that hucMSC‐Ex treatment leads to loss of NF‐κB signaling pathway through the inhibition of neddylation in vivo and in vitro, thereby alleviating inflammation.

miRNAs are a class of endogenous noncoding single‐stranded small RNAs that are involved in regulating the expression of the gene of interest at the post‐transcriptional level. The expression levels of miRNAs in different tissues and different developmental stages are significantly different, further indicating that miRNAs play an important role in regulating gene expression. miRNAs regulate many physiological processes, including early development,^50^ cell proliferation and differentiation,^51^ cell death,^52^ inflammation, immune response, and fat metabolism. Studies have shown that many miRNAs play a regulatory role in immune cells, including monocytes/macrophages, DCs, natural killer (NK) cells, mast cells, and so on, and thus participate in the development of IBD. Highly expressed miR‐21 in intestinal epithelial cells promotes Akt phosphorylation and inhibits PTEN expression, leading to damage to the intestinal mucosa^53^; miR‐141 inhibits leukocyte migration by downregulating the chemokine CKCL12β, thereby alleviating TNBS‐induced colitis.^54^ The NF‐κB family contains heterodimer transcription factors, which are mainly responsible for inflammatory response and cell survival, and play a leading role in the signal pathway of neddylation. Studies have shown that miR‐122 relieves intestinal mucosal damage by regulating the NF‐κB signaling pathway.^55^ Exosomes can secrete abundant miRNAs. In this study, hucMSC‐Ex and HFL1‐Ex were sequenced and compared, and miRNAs that target NEDD8 were selected. Verification of hucMSC‐Ex extracted from different batches showed that miR‐326 was stably and highly expressed in hucMSC‐Ex. There was no significant difference in the expression level of miR‐326 in FHC after LPS stimulation, which may be due to lower basal expression level of miR‐326 in FHC. In accordance with the predicted target, a corresponding luciferase reporter gene vector was constructed, and miR‐326 identified as a key molecule targeting NEDDB. In subsequent experiments, FHC was transfected with miR‐326 mimics and inhibitors. Cells in the mimics group significantly overexpressed miR‐326, while this expression was reduced in the inhibitor group. Suppression is associated with lower basal expression levels of miR‐326 in FHC. Under LPS stimulation, the levels of NEDD8 and cullin 1 were significantly reduced in cells that overexpressed miR‐326, the degree of binding between NEDD8 and cullin 1 was suppressed, and the expression levels of related enzymes decreased. At the same time, the CRL substrate IκB accumulated and inhibited the activation of the NF‐κB signaling pathway.

To verify the reparative effect of miR‐326 in IBD mice, the study transfected hucMSC‐Ex with miR‐326 mimics and inhibitor to establish miR‐326 high/low expression. Since miR‐326 cannot be injected directly into mice, hucMSC‐Ex acted as corresponding carriers, with results showing that hucMSC‐Ex carrying miR‐326 relieves IBD by inhibiting neddylation of cullin 1.

## 5 CONCLUSION

HucMSC‐Ex homes to damaged colorectal tissues to relieve IBD in mice. In the process of alleviating inflammation, it inhibits the binding of NEDD8 to the substrate protein cullin 1, the expression of E1, E2, and E3 enzymes in the neddylation process, as well as the activation of NF‐κB signaling pathway. HucMSC‐Ex is rich in miR‐326 which targets the expression of NEDD8 to inhibit the neddylation process and achieve the effect of relieving IBD.

## 6 STUDY HIGHLIGHTS

Findings in this research add up to knowledge on the regulatory effect of hucMSC‐Ex in intestinal mucosal inflammation, as it modulates cullin1 neddylation. miR‐326, an active cargo in hucMSC‐Ex, targets NEDD8 and prevents it from binding to cullin 1. CRL/NF‐κB signaling pathway is a classic pathway in neddylation, and loss of its transduction signal inhibits the synthesis and production of related inflammatory factors. MLN4924 is currently used as a clinical antitumor drug and has not been applied in the treatment of inflammatory damage. HucMSC‐Ex has no detrimental effect on target cells, avoids unwanted immune response caused by immunogenicity, hence providing a new target and strategy for future treatment of IBD.

## CONFLICT OF INTEREST

The authors declare no conflict of interest.

## Supporting information

Figure S1. Related parameters of NanoSight Nanoparticle Tracking Analyzer

Figure S2. (A) Gray‐scale scanning analysis of P‐NF‐κB in Figure 3A; (B) Gray‐scale scanning analysis of IκB, P‐IκB, NEDD8 in Figure 3A, *P < 0.05; **P < 0.01; ***P < 0.001 by ANOVA.

Figure S3. (A) Gray‐scale scanning analysis of PCNA in Figure 4B; (B) Gray‐scale scanning analysis of P‐NF‐κB in Figure 4C; (C) Gray‐scale scanning analysis of IκB, P‐IκB, NEDD8 in Figure 4C, *P < 0.05; **P < 0.01; ***P < 0.001 by ANOVA.

Figure S4. (A) Gray‐scale scanning analysis of P‐NF‐κB in Figure 6A; (B) Gray‐scale scanning analysis of IκB, P‐IκB, NEDD8 in Figure 6A, *P < 0.05; **P < 0.01; ***P < 0.001 by ANOVA.

Figure S5. (A) Gray‐scale scanning analysis of P‐NF‐κB in Figure 8A; (B) Gray‐scale scanning analysis of IκB, P‐IκB, NEDD8 in Figure 8A, *P < 0.05; **P < 0.01; ***P < 0.001 by ANOVA.

## Data Availability

The data that supports the findings of this study are available in the supplementary material of this article.

## References

[1] Owczarek D , Rodacki T , Domagała‐Rodacka R , et al. Diet and nutritional factors in inflammatory bowel diseases. World J Gastroenterol. 2016;22(3):895‐905. 26811635 10.3748/wjg.v22.i3.895 PMC4716043

[2] Andrews C , McLean MH , Durum SK . Interleukin‐27 as a novel therapy for inflammatory bowel disease: a critical review of the literature. Inflamm Bowel Dis. 2016;22(9):2255‐2264. 27243591 10.1097/MIB.0000000000000818 PMC4992429

[3] Axelrad JE , Lichtiger S , Yajnik V . Inflammatory bowel disease and cancer: the role of inflammation, immunosuppression, and cancer treatment. World J Gastroenterol. 2016;22(20):4794‐4801. 27239106 10.3748/wjg.v22.i20.4794 PMC4873872

[4] Uranga JA , López‐Miranda V , Lombó F , et al. Food, nutrients and nutraceuticals affecting the course of inflammatory bowel disease. Pharmacol Rep PR. 2016;68(4):816‐826. 27267792 10.1016/j.pharep.2016.05.002

[5] Li Z , Hu X , Zhong JF . Mesenchymal stem cells: characteristics, function, and application. Stem Cells Int. 2019;2019:8106818‐8106818. 30956675 10.1155/2019/8106818 PMC6431372

[6] Phinney DG , Prockop DJ . Concise review: mesenchymal stem/multipotent stromal cells: the state of transdifferentiation and modes of tissue repair–current views. Stem Cells. 2007;25(11):2896‐2902. 17901396 10.1634/stemcells.2007-0637

[7] Dimarino AM , Caplan AI , Bonfield TL . Mesenchymal stem cells in tissue repair. Front Immunol. 2013;4:201‐201. 24027567 10.3389/fimmu.2013.00201 PMC3761350

[8] Ulivi V , Tasso R , Cancedda R , et al. Mesenchymal stem cell paracrine activity is modulated by platelet lysate: induction of an inflammatory response and secretion of factors maintaining macrophages in a proinflammatory phenotype. Stem Cells Dev. 2014;23(16):1858‐1869. 24720766 10.1089/scd.2013.0567

[9] Bollini S , Gentili C , Tasso R , et al. The regenerative role of the fetal and adult stem cell secretome. J Clin Med. 2013;2(4):302‐327. 26237150 10.3390/jcm2040302 PMC4470151

[10] Ratajczak MZ , Kucia M , Jadczyk T , et al. Pivotal role of paracrine effects in stem cell therapies in regenerative medicine: can we translate stem cell‐secreted paracrine factors and microvesicles into better therapeutic strategies?. Leukemia. 2012;26(6):1166‐1173. 22182853 10.1038/leu.2011.389

[11] Bang C , Thum T . Exosomes: new players in cell‐cell communication. Int J Biochem Cell Biol. 2012;44(11):2060‐2064. 22903023 10.1016/j.biocel.2012.08.007

[12] Camussi G , Deregibus MC , Bruno S , et al. Exosomes/microvesicles as a mechanism of cell‐to‐cell communication. Kidney Int. 2010;78(9):838‐848. 20703216 10.1038/ki.2010.278

[13] Dignat‐George F , Boulanger CM . The many faces of endothelial microparticles. Arterioscler Thromb Vasc Biol. 2011;31(1):27‐33. 21160065 10.1161/ATVBAHA.110.218123

[14] Ribeiro MF , Zhu H , Millard RW , et al. Exosomes function in pro‐ and anti‐angiogenesis. Curr Angiogen. 2013;2(1):54‐59. 10.2174/22115528113020020001 PMC4217212 25374792

[15] Boulanger CM , Loyer X , Rautou P‐E , et al. Extracellular vesicles in coronary artery disease. Nat Rev Cardiol. 2017;14(5):259‐272. 28150804 10.1038/nrcardio.2017.7

[16] Théry C , Witwer KW , Aikawa E , et al. Minimal information for studies of extracellular vesicles 2018 (MISEV2018): a position statement of the International Society for Extracellular Vesicles and update of the MISEV2014 guidelines. J Extracell Vesicles. 2018;7(1):1535750‐1535750. 30637094 10.1080/20013078.2018.1535750 PMC6322352

[17] Sahoo S , Losordo DW . Exosomes and cardiac repair after myocardial infarction. Circ Res. 2014;114(2):333‐344. 24436429 10.1161/CIRCRESAHA.114.300639

[18] Kishore R , Khan M . More than tiny sacks: stem cell exosomes as cell‐free modality for cardiac repair. Circ Res. 2016;118(2):330‐343. 26838317 10.1161/CIRCRESAHA.115.307654 PMC4743531

[19] Maas SLN , Breakefield XO , Weaver AM . Extracellular vesicles: unique intercellular delivery vehicles. Trends Cell Biol. 2017;27(3):172‐188. 27979573 10.1016/j.tcb.2016.11.003 PMC5318253

[20] Colombo M , Raposo G , Théry C . Biogenesis, secretion, and intercellular interactions of exosomes and other extracellular vesicles. Annu Rev Cell Dev Biol. 2014;30:255‐289. 25288114 10.1146/annurev-cellbio-101512-122326

[21] Mallegol J , Van Niel G , Lebreton C , et al. T84‐intestinal epithelial exosomes bear MHC class II/peptide complexes potentiating antigen presentation by dendritic cells. Gastroenterology. 2007;132(5):1866‐1876. 17484880 10.1053/j.gastro.2007.02.043

[22] Xu AT , Lu JT , Ran ZH , et al. Exosome in intestinal mucosal immunity. J Gastroenterol Hepatol. 2016;31(10):1694‐1699. 27061439 10.1111/jgh.13413

[23] Fonseca‐Camarillo G , Yamamoto‐Furusho JK . Immunoregulatory pathways involved in inflammatory bowel disease. Inflamm Bowel Dis. 2015;21(9):2188‐2193. 26111210 10.1097/MIB.0000000000000477

[24] Zhao Y , Morgan MA , Sun Y . Targeting neddylation pathways to inactivate cullin‐RING ligases for anticancer therapy. Antioxid Redox Signaling. 2014;21(17):2383‐2400. 10.1089/ars.2013.5795 PMC4241876 24410571

[25] Enchev RI , Schulman BA , Peter M . Protein neddylation: beyond cullin‐RING ligases. Nat Rev Mol Cell Biol. 2015;16(1):30‐44. 25531226 10.1038/nrm3919 PMC5131867

[26] Godbersen JC , Humphries LA , Danilova OV , et al. The Nedd8‐activating enzyme inhibitor MLN4924 thwarts microenvironment‐driven NF‐κB activation and induces apoptosis in chronic lymphocytic leukemia B cells. Clin Cancer Res. 2014;20(6):1576‐1589. 24634471 10.1158/1078-0432.CCR-13-0987 PMC3960291

[27] Han K , Wang Q , Cao H , et al. The NEDD8‐activating enzyme inhibitor MLN4924 induces G2 arrest and apoptosis in T‐cell acute lymphoblastic leukemia. Oncotarget. 2016;7(17):23812‐23824. 26993774 10.18632/oncotarget.8068 PMC5029665

[28] Milhollen MA , Traore T , Adams‐Duffy J , et al. MLN4924, a NEDD8‐activating enzyme inhibitor, is active in diffuse large B‐cell lymphoma models: rationale for treatment of NF‐{kappa}B‐dependent lymphoma. Blood. 2010;116(9):1515‐1523. 20525923 10.1182/blood-2010-03-272567

[29] Qiao C , Xu W , Zhu W , et al. Human mesenchymal stem cells isolated from the umbilical cord. Cell Biol Int. 2008;32(1):8‐15. 17904875 10.1016/j.cellbi.2007.08.002

[30] Andreu Z , Yáñez‐Mó M . Tetraspanins in extracellular vesicle formation and function. Front Immunol. 2014;5:442‐442. 25278937 10.3389/fimmu.2014.00442 PMC4165315

[31] Lin J , Li J , Huang B , et al. Exosomes: novel biomarkers for clinical diagnosis. Sci World J. 2015;2015:657086‐657086. 10.1155/2015/657086 PMC4322857 25695100

[32] Sun D , Zhuang X , Zhang S , et al. Exosomes are endogenous nanoparticles that can deliver biological information between cells. Adv Drug Deliv Rev. 2013;65(3):342‐347. 22776312 10.1016/j.addr.2012.07.002

[33] Choi J‐Y , Kim S , Kwak H‐B , et al. Extracellular vesicles as a source of urological biomarkers: lessons learned from advances and challenges in clinical applications to major diseases. Int Neurourol J. 2017;21(2):83‐96. 28673066 10.5213/inj.1734961.458 PMC5497201

[34] Kalani A , Tyagi A , Tyagi N . Exosomes: mediators of neurodegeneration, neuroprotection and therapeutics. Mol Neurobiol. 2014;49(1):590‐600. 23999871 10.1007/s12035-013-8544-1 PMC3951279

[35] Rabinowits G , Gerçel‐Taylor C , Day JM , et al. Exosomal microRNA: a diagnostic marker for lung cancer. Clin Lung Cancer. 2009;10(1):42‐46. 19289371 10.3816/CLC.2009.n.006

[36] Vlassov AV , Magdaleno S , Setterquist R , et al. Exosomes: current knowledge of their composition, biological functions, and diagnostic and therapeutic potentials. Biochim Biophys Acta. 2012;1820(7):940‐948. 22503788 10.1016/j.bbagen.2012.03.017

[37] Leoni G , Neumann P‐A , Kamaly N , et al. Annexin A1‐containing extracellular vesicles and polymeric nanoparticles promote epithelial wound repair. J Clin Invest. 2015;125(3):1215‐1227. 25664854 10.1172/JCI76693 PMC4362251

[38] Yang X , Meng S , Jiang H , et al. Exosomes derived from interleukin‐10‐treated dendritic cells can inhibit trinitrobenzene sulfonic acid‐induced rat colitis. Scand J Gastroenterol. 2010;45(10):1168‐1177. 20469967 10.3109/00365521.2010.490596

[39] Wang L , Yu Z , Wan S , et al. Exosomes derived from dendritic cells treated with schistosoma japonicum soluble egg antigen attenuate DSS‐induced colitis. Front Pharmacol. 2017;8:651‐651. 28959207 10.3389/fphar.2017.00651 PMC5603738

[40] Eichenberger RM , Ryan S , Jones L , et al. Hookworm secreted extracellular vesicles interact with host cells and prevent inducible colitis in mice. Front Immunol. 2018;9:850‐850. 29760697 10.3389/fimmu.2018.00850 PMC5936971

[41] Chen Y , Shao J‐Z , Xiang L‐X , et al. Mesenchymal stem cells: a promising candidate in regenerative medicine. Int J Biochem Cell Biol. 2008;40(5):815‐820. 18295530 10.1016/j.biocel.2008.01.007

[42] Sadeghi A , Rostamirad A , Seyyedebrahimi S , et al. Curcumin ameliorates palmitate‐induced inflammation in skeletal muscle cells by regulating JNK/NF‐kB pathway and ROS production. Inflammopharmacology. 2018;26(5):1265‐1272. 29644554 10.1007/s10787-018-0466-0

[43] Rashidian A , Muhammadnejad A , Dehpour A‐R , et al. Atorvastatin attenuates TNBS‐induced rat colitis: the involvement of the TLR4/NF‐kB signaling pathway. Inflammopharmacology. 2016;24(2‐3):109‐118. 27038922 10.1007/s10787-016-0263-6

[44] Muniandy K , Gothai S , Badran KMH , et al. Suppression of proinflammatory cytokines and mediators in LPS‐induced RAW 264.7 macrophages by stem extract of alternanthera sessilis via the inhibition of the NF‐κB pathway. J Immunol Res. 2018;2018:3430684‐3430684. 30155492 10.1155/2018/3430684 PMC6093060

[45] Schwechheimer C . NEDD8‐its role in the regulation of Cullin‐RING ligases. Curr Opin Plant Biol. 2018;45(Pt A):112‐119. 29909289 10.1016/j.pbi.2018.05.017

[46] Ehrentraut SF , Curtis VF , Wang RX , et al. Perturbation of neddylation‐dependent NF‐κB responses in the intestinal epithelium drives apoptosis and inhibits resolution of mucosal inflammation. Mol Biol Cell. 2016;27(23):3687‐3694. 10.1091/mbc.E16-05-0273 PMC5170552 27682585

[47] Lee I , Schindelin H . Structural insights into E1‐catalyzed ubiquitin activation and transfer to conjugating enzymes. Cell. 2008;134(2):268‐278. 18662542 10.1016/j.cell.2008.05.046

[48] Huang G , Kaufman AJ , Ramanathan Y , et al. SCCRO (DCUN1D1) promotes nuclear translocation and assembly of the neddylation E3 complex. J Biol Chem. 2011;286(12):10297‐10304. 21247897 10.1074/jbc.M110.203729 PMC3060484

[49] Nawrocki ST , Griffin P , Kelly KR , et al. MLN4924: a novel first‐in‐class inhibitor of NEDD8‐activating enzyme for cancer therapy. Expert Opin Investig Drugs. 2012;21(10):1563‐1573. 10.1517/13543784.2012.707192 22799561

[50] Reinhart BJ , Slack FJ , Basson M , et al. The 21‐nucleotide let‐7 RNA regulates developmental timing in *Caenorhabditis elegans* . Nature. 2000;403(6772):901‐906. 10706289 10.1038/35002607

[51] Kawasaki H , Taira K . Retraction: hes1 is a target of microRNA‐23 during retinoic‐acid‐induced neuronal differentiation of NT2 cells. Nature. 2003;426(6962):100‐100. 14603326 10.1038/nature02141

[52] Brennecke J , Cohen SM . Towards a complete description of the microRNA complement of animal genomes. Genome Biol. 2003;4(9):228‐228. 12952528 10.1186/gb-2003-4-9-228 PMC193649

[53] Zhang B , Wang M , Gong A , et al. HucMSC‐Exosome Mediated‐Wnt4 signaling is required for cutaneous wound healing. Stem Cells. 2015;33(7):2158‐2168. 24964196 10.1002/stem.1771

[54] Huang Z , Shi T , Zhou Q , et al. miR‐141 regulates colonic leukocytic trafficking by targeting CXCL12β during murine colitis and human Crohn's disease. Gut. 2014;63(8):1247‐1257. 24000293 10.1136/gutjnl-2012-304213

[55] Chen Y , Wang C , Liu Y , et al. miR‐122 targets NOD2 to decrease intestinal epithelial cell injury in Crohn's disease. Biochem Biophys Res Commun. 2013;438(1):133‐139. 23872065 10.1016/j.bbrc.2013.07.040

