# Supplementary figures and images for: HucMSC‐exosomes carrying miR‐326 inhibit neddylation to relieve inflammatory bowel disease in mice
Source: Clin Transl Med. 2020 Jun 21;10(2):e113. doi: 10.1002/ctm2.113 (PMC7403704; doi:10.1002/ctm2.113)

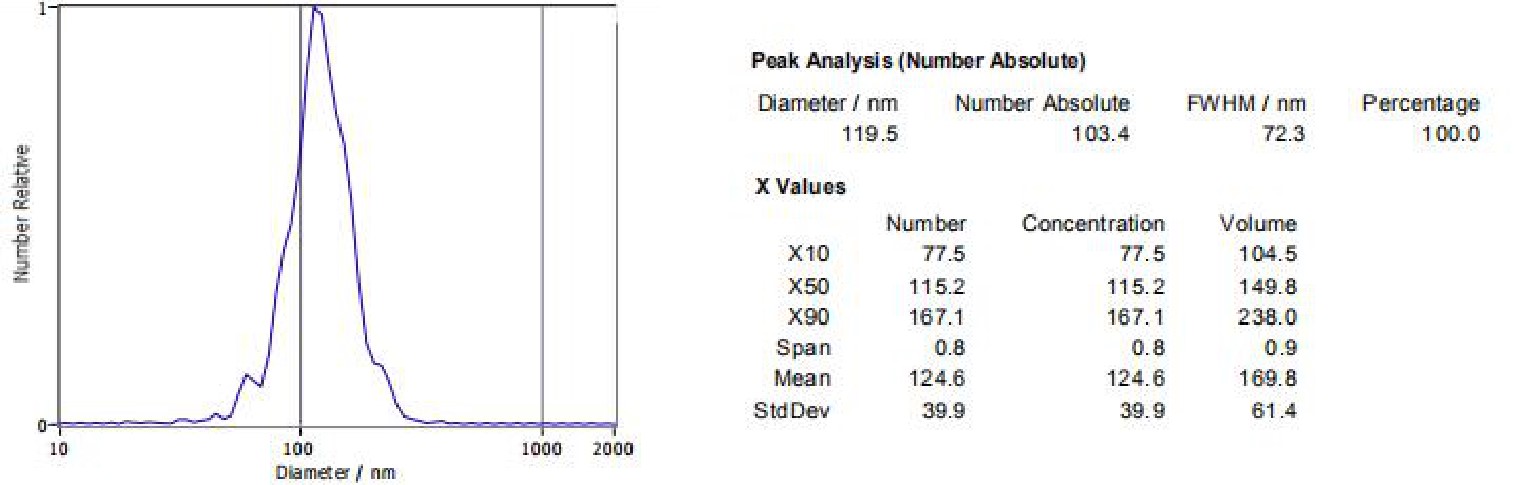

Supplement: Supplementary file 1 — Figure S1. Related parameters of NanoSight Nanoparticle Tracking Analyzer [file CTM2-10-e113-s001.jpg]

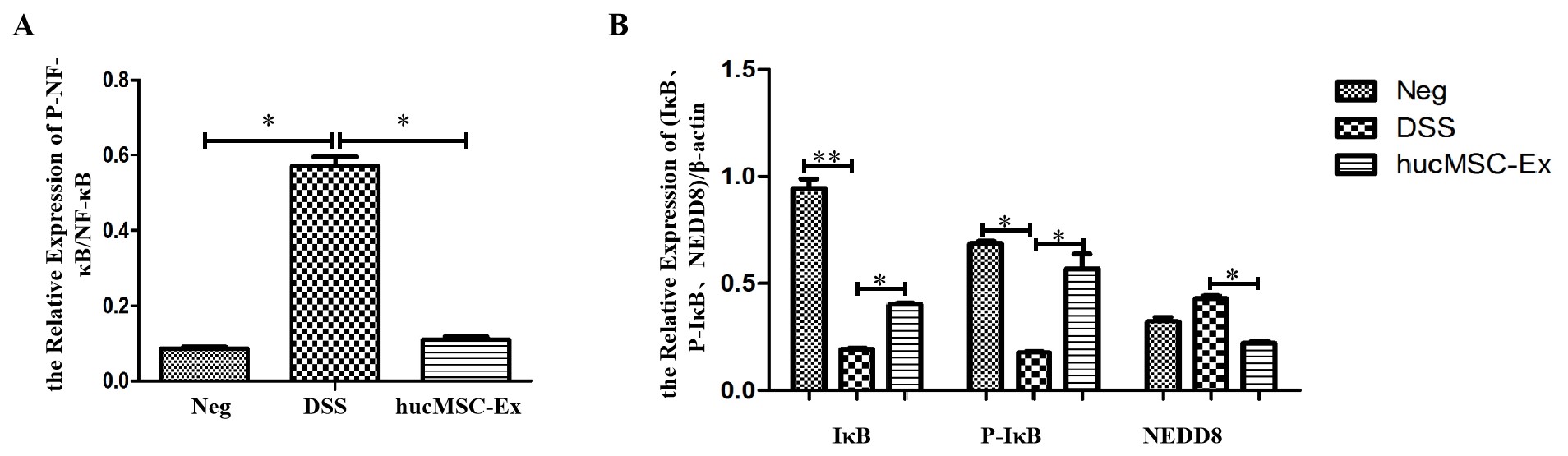

Supplement: Supplementary file 2 — Figure S2. (A) Gray‐scale scanning analysis of P‐NF‐κB in Figure 3A; (B) Gray‐scale scanning analysis of IκB, P‐IκB, NEDD8 in Figure 3A, *P < 0.05; **P < 0.01; ***P < 0.001 by ANOVA. [file CTM2-10-e113-s002.jpg]

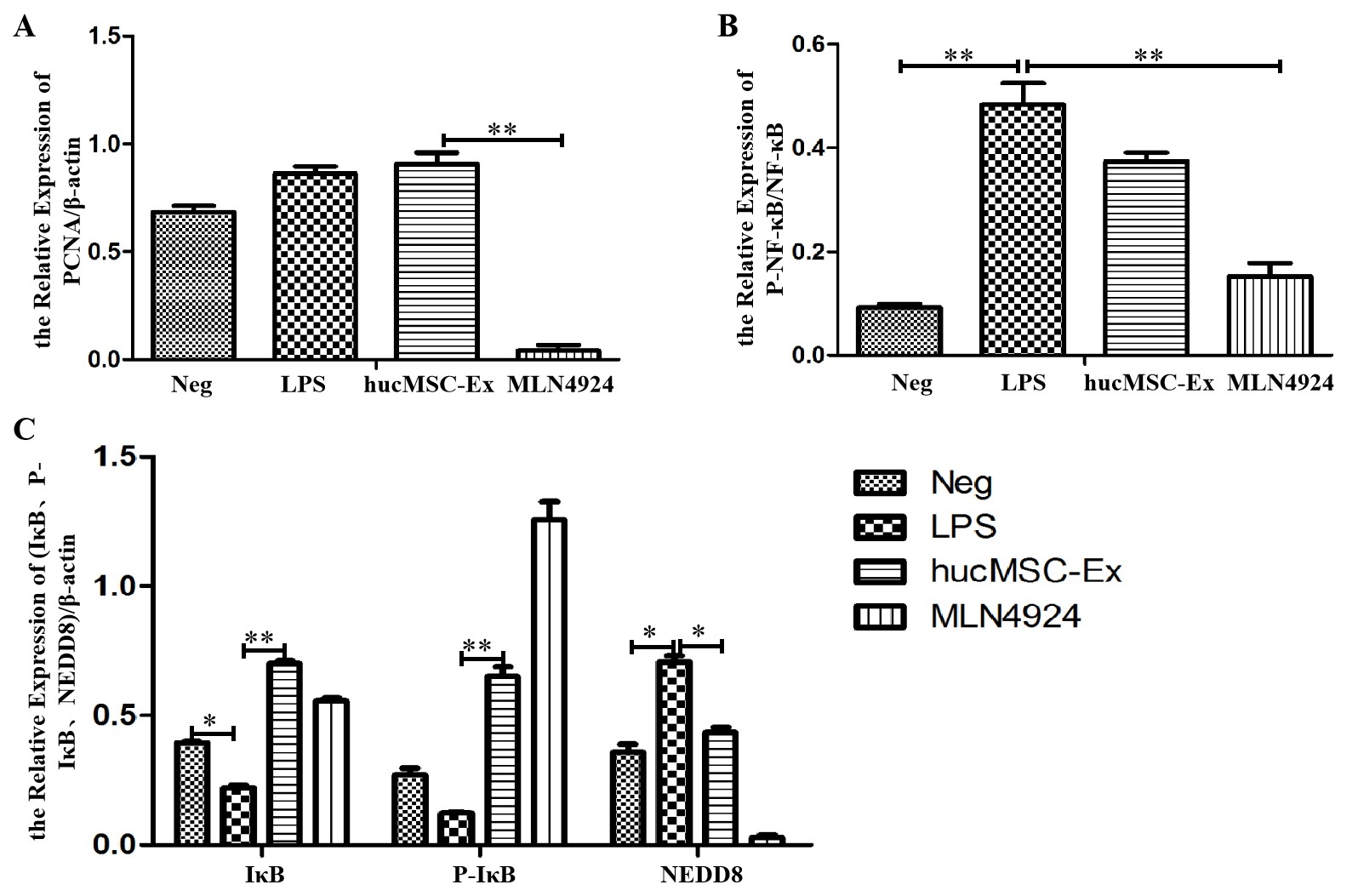

Supplement: Supplementary file 3 — Figure S3. (A) Gray‐scale scanning analysis of PCNA in Figure 4B; (B) Gray‐scale scanning analysis of P‐NF‐κB in Figure 4C; (C) Gray‐scale scanning analysis of IκB, P‐IκB, NEDD8 in Figure 4C, *P < 0.05; **P < 0.01; ***P < 0.001 by ANOVA. [file CTM2-10-e113-s003.jpg]

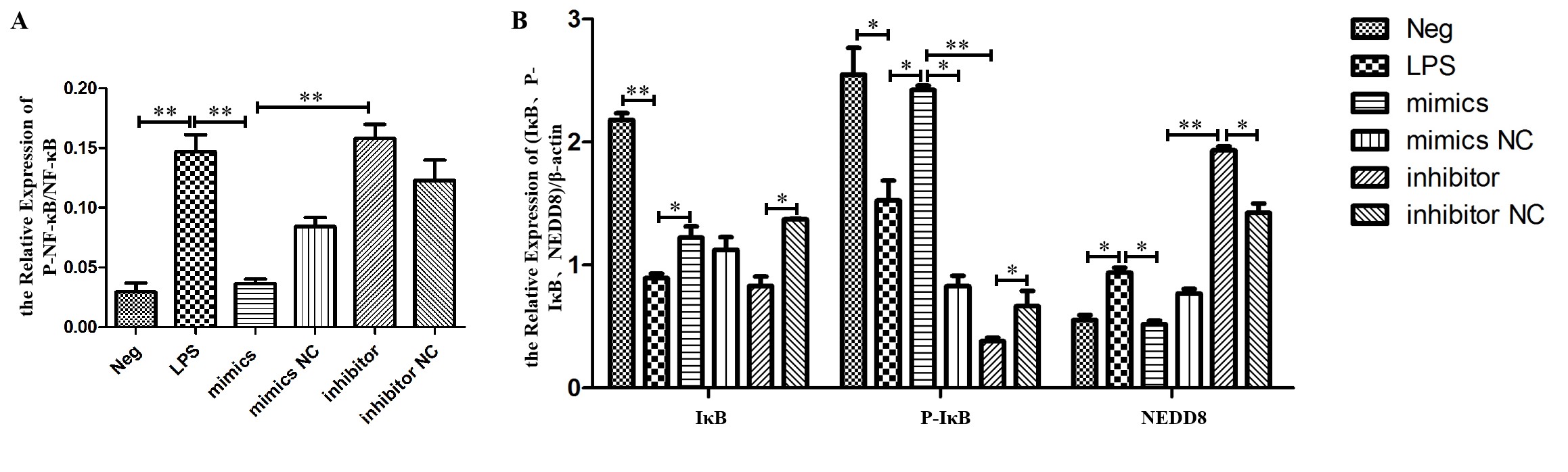

Supplement: Supplementary file 4 — Figure S4. (A) Gray‐scale scanning analysis of P‐NF‐κB in Figure 6A; (B) Gray‐scale scanning analysis of IκB, P‐IκB, NEDD8 in Figure 6A, *P < 0.05; **P < 0.01; ***P < 0.001 by ANOVA. [file CTM2-10-e113-s004.jpg]

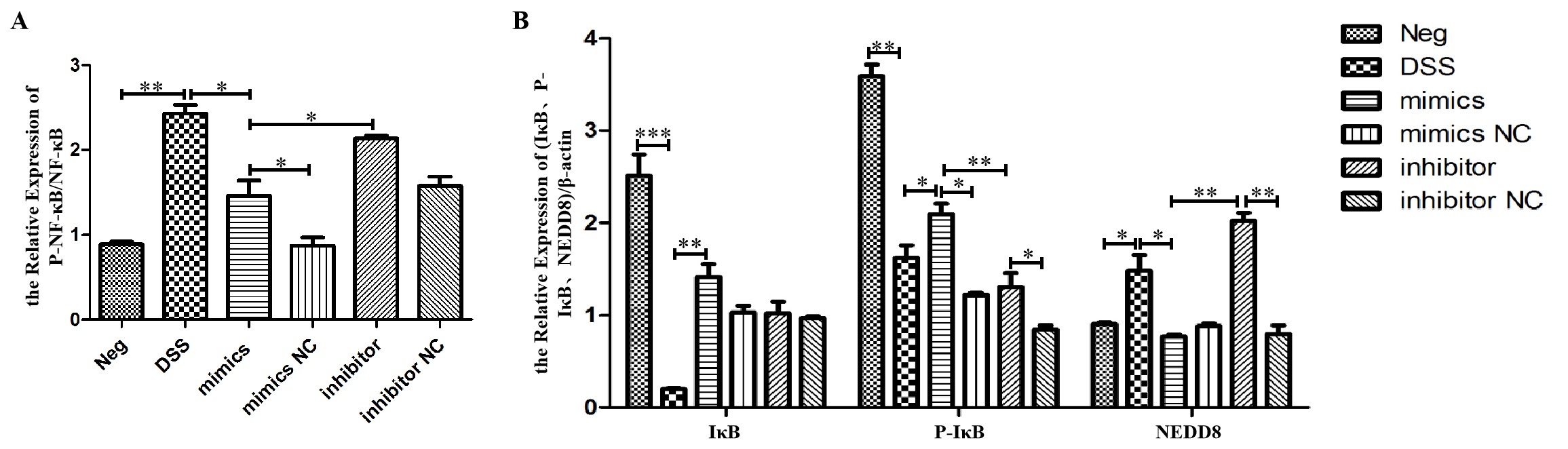

Supplement: Supplementary file 5 — Figure S5. (A) Gray‐scale scanning analysis of P‐NF‐κB in Figure 8A; (B) Gray‐scale scanning analysis of IκB, P‐IκB, NEDD8 in Figure 8A, *P < 0.05; **P < 0.01; ***P < 0.001 by ANOVA. [file CTM2-10-e113-s005.jpg]
